# Harnessing Artificial Intelligence to Deliver Growth Mindset Education in a Pre-matriculation Curriculum for Incoming Medical Students

**DOI:** 10.7759/cureus.106023

**Published:** 2026-03-28

**Authors:** Troy Weinstein, Claire Pishko, Derek Honeyman, Claudio Cheuquen, Katie Kowalek, Daniel C Butler, Elaine Situ-LaCasse, Richard Amini

**Affiliations:** 1 Medical School, University of Arizona College of Medicine - Tucson, Tucson, USA; 2 Medical Education, University of Arizona College of Medicine - Tucson, Tucson, USA; 3 Pediatrics, University of Arizona College of Medicine - Tucson, Tucson, USA; 4 Dermatology, University of Arizona College of Medicine - Tucson, Tucson, USA; 5 Emergency Medicine, University of Arizona College of Medicine - Tucson, Tucson, USA

**Keywords:** artificial intelligence, chatgpt, growth mindset, large language models, medical education, resilience

## Abstract

Background: Growth mindset, the belief that intelligence and abilities can be developed through effort and strategy, underpins resilience and adaptive learning in the demanding context of medical education. Yet the intensity of medical curricula leaves little room for supplemental content, even when it addresses essential skills like resilience and adaptability. Emerging tools such as large language models (LLMs) now enable faculty to efficiently generate accessible, learner-centered educational materials that support scalable delivery of growth mindset content.

Objective: The objective of this study is to test the feasibility, engagement, and impact of “Mindset Mondays,” a 10-week email and podcast curriculum for incoming medical students.

Methods: Medical students at the University of Arizona College of Medicine - Tucson received weekly emails during the pre-matriculation period prior to the start of formal medical school. These emails featured thematic summaries of growth mindset concepts, key takeaways, quotes, and reflection prompts. Extended PDF summaries and 10-15 minute podcasts supplemented each email. Surveys assessed demographics, engagement, and retrospective pre/post growth mindset beliefs via Likert-based surveys.

Results: Of 94 respondents, 76.6% were first-generation students and 51.1% reported prior growth mindset exposure. Students engaged with an average of 4.05 emails (SD = 3.28), 2.60 PDFs (SD = 2.89), and 1.03 podcasts (SD = 2.19). Composite growth mindset scores improved significantly (Pre: M = 4.92, SD = 0.61; Post: M = 5.17, SD = 0.60; *p* < 0.001; Cohen’s d_z_ = 0.71). Seven out of eight Likert items showed significant improvement (*p* < 0.05; effect sizes 0.32-0.46). Greater engagement was associated with greater improvement across all formats (r = .339-.399, p < .001).

Conclusion: Delivering growth mindset education through LLM-assisted emails and podcasts was feasible, scalable, and associated with small but significant self-reported growth mindset gains. Early, structured exposure to growth mindset principles may strengthen resilience, promote receptivity to feedback, and better prepare students for the demands of medical school.

## Introduction

Medical students enter training with diverse educational backgrounds, study habits, and coping strategies, yet few receive explicit instruction on resilience and adaptive learning [1,2]. The growth mindset, defined as the belief that intelligence and abilities can be developed through effort, persistence, and feedback, has been associated with improved motivation, resilience, and long-term performance in educational settings [3]. In contrast, fixed mindset beliefs, which view intelligence as static, are linked to avoidance of challenges, reduced receptivity to feedback, and vulnerability to stress and burnout [3]. For learners entering the high-stakes and feedback-rich environment of medical school, cultivating a growth mindset may provide an essential foundation for academic success and well-being [4,5].

Carol Dweck’s* Mindset: The New Psychology of Success* remains a seminal text introducing these principles, but the demands of medical training leave little time for students to read and digest its content [3]. Mindset interventions have demonstrated effectiveness in K-12 and undergraduate populations, with meta-analyses confirming small but meaningful effects on academic outcomes [4-7]. However, scalable and replicable applications within medical education remain limited [1,5].

Pre-matriculation represents a critical window for intervention. During this period, students are highly motivated, unburdened by coursework, and receptive to strategies that may ease their transition. Early exposure to growth mindset principles may normalize adaptive responses to feedback, strengthen persistence in the face of setbacks, and equip learners with tools to manage the challenges of medical school [4,6,8,9]. However, delivering this material in a way that is both scalable and engaging has been a persistent challenge.

Large language models (LLMs) present a novel solution. These tools can rapidly generate accurate content distilled from lengthy source materials [10,11]. By leveraging LLMs, educators can transform key principles from the growth mindset literature into concise, multimodal resources such as emails, podcasts, and summaries that fit naturally into the rhythms of student life.

The objective of this single-institution study was to evaluate the feasibility, engagement, and associated growth mindset changes after implementing “Mindset Mondays,” a pre-matriculation curriculum delivered to incoming medical students. The primary outcome was change in composite self-reported growth mindset score measured using an eight-item Likert-based instrument. Secondary outcomes included associations between engagement with curriculum components and changes in mindset scores.

We hypothesized that participation in the “Mindset Mondays” curriculum would be associated with directional increases in growth-oriented beliefs, including beliefs about the malleability of intelligence, persistence in the face of challenge, and adaptive responses to feedback. We further hypothesized that greater engagement with the curriculum would be positively associated with improvement in composite growth mindset scores. This is the first study to evaluate a pre-matriculation program focused on a growth mindset that was developed and delivered with the assistance of LLMs.

## Materials and methods

This study used a single-group, cross-sectional design with retrospective pre/post self-assessment to evaluate feasibility, engagement, and perceived changes in growth mindset beliefs. This study was reviewed by the University of Arizona Institutional Review Board (Submission ID: STUDY00006844) and approved as a Quality Improvement (QI) study, which does not constitute human subjects research.

Emails were sent to all 120 accepted medical students matriculating in July 2025 at the University of Arizona College of Medicine-Tucson. Each message included an option to opt out of further emails. The intervention period was May 25, 2025, through July 21, 2025, during which 10 weekly “Mindset Mondays” emails were distributed. Participation in reviewing the emails, PDFs, and podcasts was voluntary. The survey was administered during July orientation. Inclusion criteria consisted of completing all survey items in Qualtrics. Students who did not submit a fully completed survey were excluded. A total of 94 students (78.3 percent) met the inclusion criteria, and no additional exclusion criteria were applied.

The intervention consisted of a 10-week series of pre-matriculation emails delivered each Monday during the summer prior to matriculation. Each email included a motivational quote, an original thematic summary of growth mindset concepts, three key takeaways emphasizing practical application for medical students, reflective prompts to encourage self-assessment, and a lighthearted “joke of the week.” To accommodate different learning preferences, each email also included an extended two-page PDF conceptual summary and a 10-14-minute podcast episode reviewing the same conceptual content.

LLMs were used to assist in the development of these materials. ChatGPT (OpenAI, San Francisco, United States) was employed to support synthesis of established growth mindset constructs described in the peer-reviewed literature, and NotebookLM (Google Labs, Mountain View, United States) was used to generate podcast scripts and audio content. AI-assisted content was developed through an iterative process of synthesis and faculty refinement rather than standardized or fixed prompts, which limits exact reproducibility. All AI-assisted outputs were reviewed and edited by the senior author to ensure accuracy, appropriateness, and alignment with educational objectives. No copyrighted text from *Mindset: The New Psychology of Success* or any other proprietary source was reproduced, adapted, or translated; therefore, permission from the publisher or copyright holder was not required.

At in-person orientation, students completed a survey (Appendix) that included demographic items (first-generation medical student status, prior exposure to growth mindset instruction, and path to medical school), engagement measures (number of emails read, PDF summaries reviewed, and podcasts listened to), and a retrospective pre/post growth mindset assessment. The mindset instrument consisted of eight Likert-scale items (1 = strongly disagree, 6 = strongly agree) informed conceptually by validated growth-mindset surveys. Survey items were independently written by the study team to assess growth- and fixed-mindset constructs as defined in the social-cognitive literature on implicit theories of intelligence [3,4,6]. Open-ended reflection items were also included to capture qualitative feedback on the perceived usefulness and relevance of the curriculum and were analyzed descriptively without formal qualitative methods.

Descriptive statistics were calculated for demographics and engagement. Paired-sample t-tests compared retrospective pre- and post-intervention mindset scores, with Cohen’s d_z_ reported as an estimate of effect size. Internal consistency of the mindset scale was assessed using Cronbach’s α. Subgroup analyses used independent-sample t-tests for first-generation status and prior growth mindset exposure, and a one-way ANOVA for path to medical school. Pearson correlation coefficients assessed associations between engagement (emails, PDFs, and podcasts) and changes in mindset scores. All statistical tests were two-sided, and significance was defined as p < 0.05.

## Results

A total of 94 (78.3%) incoming medical students completed the survey. Of these, 72 (76.6%) identified as first-generation medical students and 22 (23.4%) did not. Forty-eight students (51.1%) reported prior formal instruction in growth mindset, while 46 (48.9%) reported no such exposure. Regarding the pathway to medical school, 21 students (22.3%) matriculated directly from undergraduate studies, 40 (42.6%) reported one to two gap years, 27 (28.7%) reported more than two gap years, and six (6.4%) identified as career changers (Table 1).

**Table 1:** Participant demographics of the 2025 entering class (n = 94)

Characteristic	n (%)
Total respondents	94 (78.3 %)
First-generation medical student	72 (76.6 %)
Prior growth-mindset instruction	48 (51.1 %)
Path to medical school	
Direct from undergraduate studies	21 (22.3 %)
1–2 gap years	40 (42.6 %)
> 2 gap years	27 (28.7 %)
Career changer	6 (6.4 %)

Engagement with the intervention varied. Students reported reading an average of 4.05 Mindset Monday emails (SD = 3.28), reviewing 2.6 PDF chapter summaries (SD = 2.89), and listening to 1.03 podcasts (SD = 2.19). Engagement results are presented in Table 2.

**Table 2:** Engagement with the “Mindset Monday” content

Engagement Measure	Mean (SD)
Emails read (0–10)	4.05 (3.28)
PDFs reviewed (0–10)	2.60 (2.89)
Podcasts listened (0–10)	1.03 (2.19)

Composite growth mindset scores were calculated by averaging responses across the eight Likert-scale items for each participant, with higher values reflecting stronger growth mindset beliefs. Scores increased from a mean of 4.92 (SD = 0.61) before the intervention to 5.17 (SD = 0.60) after the intervention (p < 0.001). The effect size was Cohen’s d_z_ = 0.71, indicating a medium-large effect size. Seven out of eight individual mindset items showed statistically significant improvement, with item-level effect sizes ranging from 0.32 to 0.46. Internal consistency was strong for both administrations of the instrument (Cronbach’s α = 0.784 pre-intervention, 0.785 post-intervention). These values indicate good internal reliability of the eight-item scale across administrations (Table 3). These changes occurred despite high baseline growth mindset scores, suggesting that observable improvements were constrained by limited headroom for change.

**Table 3:** Pre- and post-intervention growth mindset scores p-values were generated using two-sided paired-sample t-tests. Cohen’s d_z_ represents the effect size.

Question	Pre-Mean (SD)	Post-Mean (SD)	p	Cohen’s d_z_
You have a certain amount of intelligence, and you really can’t do much to change it. (reverse)	4.94 (1.18)	5.03 (1.22)	0.267	0.12
You can always substantially change how intelligent you are.	4.60 (1.03)	4.90 (1.04)	< 0.001	0.37
Learning is more important to me than getting perfect grades.	4.87 (1.05)	5.12 (0.95)	0.002	0.32
I see setbacks as opportunities to learn and grow.	4.97 (1.05)	5.35 (0.79)	< 0.001	0.46
If I can’t do something right away, I usually give up. (reverse)	4.84 (0.98)	5.14 (1.16)	< 0.001	0.37
When something is difficult, I try to find new strategies to succeed.	5.23 (0.63)	5.38 (0.55)	0.002	0.32
I seek out feedback to help me grow, even when it’s critical.	4.99 (0.78)	5.26 (0.75)	< 0.001	0.41
I try to learn from criticism rather than take it personally.	4.91 (0.91)	5.19 (0.91)	< 0.001	0.45
Composite Mindset Score	4.92 (0.61)	5.17 (0.60)	< 0.001	0.71

Subgroup analyses found no significant differences in mindset score change based on first-generation status (p = 0.370), prior growth mindset instruction (p = 0.219), or path to medical school (p = 0.339).

Greater engagement with the materials was positively correlated with greater improvement in mindset scores. The number of emails read (r = 0.365, p < 0.001), PDFs reviewed (r = 0.399, p < 0.001), and podcasts listened to (r = 0.339, p < 0.001) were each significantly correlated with composite mindset change scores. Qualitative reflections and perceived-impact responses supported these findings, with many students noting that the curriculum increased their confidence and preparedness for medical school.

## Discussion

This study evaluated the feasibility and outcomes of a pre-matriculation curriculum designed to introduce growth mindset principles to incoming medical students. Using weekly emails, PDF summaries, and podcasts generated with the assistance of LLMs and NotebookLM, the curriculum was associated with significant self-reported improvement in the composite growth mindset score, with seven of eight individual items showing statistically significant pre/post differences, and greater engagement corresponding to greater gains. Importantly, these associated improvements were observed regardless of prior exposure to formal growth mindset training, supporting the idea that brief, scalable interventions may be associated with reinforcement of learning beliefs [9,12]. The positive correlations between engagement and score improvement suggest an association between participation and observed gains.

The small but significant effect observed in this study aligns with meta-analyses showing that growth mindset interventions generally yield modest improvements in academic outcomes, with the greatest benefits among students from disadvantaged or academically at-risk backgrounds [5,7]. Most participants (76.6%) in the present study identified as first-generation medical students, underscoring the relevance of equitable interventions in diverse cohorts. However, we did not observe a stronger association among first-generation students, contrasting with findings from Canning et al., in which instructor-delivered mindset messages particularly benefited first-generation learners [13]. This difference may reflect the variation in delivery format, with our self-directed, asynchronous pre-matriculation model differing from their instructor-led, classroom-based intervention that allowed immediate reinforcement of key concepts.

First-generation students, having already navigated significant academic and personal barriers, may possess well-developed resilience that limits the relative impact of additional mindset training. Nonetheless, students in our cohort reported measurable improvements, suggesting that early exposure to mindset principles offers a valuable foundation for medical training. Delivering the intervention before matriculation capitalizes on a period of high motivation and low curricular burden [8,9]. Prior work similarly shows that structured pre-matriculation programs enhance early academic performance and predict first-year success, particularly among students from underrepresented or academically disadvantaged backgrounds [4,8]. Collectively, these findings support pre-matriculation as an optimal window for scalable interventions that promote resilience, equity, and readiness for the challenges of medical school.

Growth mindset principles are highly relevant in medical education, where students face demanding workloads, frequent evaluations, and clinical uncertainty [14,15]. As outlined in the growth mindset literature, even small shifts in how learners perceive their ability to grow have been associated with increased motivation, persistence, and openness to feedback [3]. Brief psychological interventions that reframe challenges as opportunities have shown lasting effects on resilience and professional identity formation [9]. In our cohort, improvements in persistence and feedback receptivity aligned with these studies, suggesting that early exposure to growth mindset concepts may support the development of adaptive learning attitudes during the transition into medical training.

The use of ChatGPT and NotebookLM in content creation represents a novel and scalable approach to medical education [10,11]. Faculty were able to transform established growth mindset concepts into multimodal resources that were concise, engaging, and tailored to medical students. Importantly, LLMs served as supportive tools that facilitated efficient development of educational materials rather than acting as individual drivers of observed change [10,11]. The positive association between engagement and mindset improvement highlights the potential of this model to deliver interventions at scale with limited faculty burden.

This study has several limitations. It was conducted at a single institution using a single-group, cross-sectional design with retrospective pre/post self-assessment, which limits causal inference and definitive attribution of observed changes to the intervention. The retrospective format is susceptible to recall and response-shift bias, and outcomes were self-reported rather than objective. Long-term academic or professional outcomes were not assessed. Baseline growth mindset scores were high, introducing potential ceiling effects that constrained the magnitude of observable change. Because the survey was study-developed and AI-assisted content was generated iteratively rather than via fixed prompts, exact replication of the intervention materials is limited. Future prospective, controlled, and multi-institutional studies are needed to more rigorously evaluate the durability and causal impact of pre-matriculation growth mindset interventions.

## Conclusions

The “Mindset Mondays” curriculum demonstrated the feasibility of delivering growth mindset principles to incoming medical students through a brief, multimodal, pre-matriculation program. Students engaged with emails, PDFs, and podcasts developed with the assistance of ChatGPT and NotebookLM demonstrated small but statistically significant self-reported improvements in growth mindset scores. Importantly, these associations were observed regardless of first-generation status, prior mindset instruction, or pathway to medical school, highlighting the broad accessibility of this model.

Introducing resilience-oriented content before the start of formal coursework may offer an opportunity to reach students during a period of high motivation and low curricular burden, providing students with practical strategies to approach feedback, setbacks, and academic challenges. LLMs assisted faculty in generating high-quality content rapidly, supporting scalability and innovation in medical education. Future work should prospectively evaluate the long-term impact of pre-matriculation growth mindset interventions on academic performance, professional identity formation, and well-being, and explore the potential for broader adoption across institutions.

## Appendices

The complete survey instrument is provided below and is organized by survey section, including background information, growth mindset belief items, and perceived impact. This survey was developed by the study team for this project and was conceptually adapted from established growth-mindset frameworks described in prior literature.

Growth mindset curriculum survey

Welcome to the Growth Mindset Curriculum Survey

This brief survey is part of a Quality Improvement effort to enhance orientation programming at our medical school. Your responses will help us improve future mindset-based wellness interventions for incoming students.

This survey is in reference to the 10-week email series entitled Mindset Mondays.

All answers are anonymous and will be used only in aggregate for analysis. There are no right or wrong responses - please answer honestly based on your own experience.

**Table 4:** Background information

Question	Response Options
1. What was your path to medical school?	Direct from undergraduate studies
	1–2 gap years
	More than 2 gap years
	Career changer
2. Are you the first in your family to attend medical school?	Yes
	No
3. Have you received formal instruction on growth mindset before this curriculum?	Yes
	No
4. How many Mindset Monday materials did you complete?	Emails: 0–10
	PDF summaries: 0–10
	Podcasts: 0–10

**Table 5:** Mindset beliefs before the curriculum Response options range from 1 to 6, corresponding to Strongly Disagree, Disagree, Slightly Disagree, Slightly Agree, Agree, and Strongly Agree.

Statement	Response Scale
You have a certain amount of intelligence, and you really can’t do much to change it. (reverse-scored)	1–6
You can always substantially change how intelligent you are.	1–6
Learning is more important to me than getting perfect grades.	1–6
I see setbacks as opportunities to learn and grow.	1–6
If I can’t do something right away, I usually give up. (reverse-scored)	1–6
When something is difficult, I try to find new strategies to succeed.	1–6
I seek out feedback to help me grow, even when it’s critical.	1–6
I try to learn from criticism rather than take it personally.	1–6

**Table 6:** Mindset beliefs after the curriculum Response options range from 1 to 6, corresponding to Strongly Disagree, Disagree, Slightly Disagree, Slightly Agree, Agree, and Strongly Agree.

Statement	Response Scale
You have a certain amount of intelligence, and you really can’t do much to change it. (reverse-scored)	1–6
You can always substantially change how intelligent you are.	1–6
Learning is more important to me than getting perfect grades.	1–6
I see setbacks as opportunities to learn and grow.	1–6
If I can’t do something right away, I usually give up. (reverse-scored)	1–6
When something is difficult, I try to find new strategies to succeed.	1–6
I seek out feedback to help me grow, even when it’s critical.	1–6
I try to learn from criticism rather than take it personally.	1–6

**Table 7:** Perceived impact of the curriculum Response options range from 1 to 6, corresponding to Strongly Disagree, Disagree, Slightly Disagree, Slightly Agree, Agree, and Strongly Agree.

Statement	Response Scale
I believe the Mindset emails and content will better equip me to handle the challenges of medical school.	1–6
I plan to apply the growth mindset principles I learned during medical school.	1–6
The Mindset Mondays curriculum helped me feel less intimidated by the idea of failing or struggling in medical school.	1–6
I see challenges in medical school as opportunities for growth rather than threats to my self-worth.	1–6
Briefly describe one way your mindset has changed — or one thing you plan to do differently — because of what you learned in this curriculum.	Open-ended response
